# A158 DEVELOPMENT OF MODELS TO SUDY THE INTERACTION BETWEEN CANCER STEM CELLS AND THE MICROENVIRONMENT IN COLON CANCER

**DOI:** 10.1093/jcag/gwad061.158

**Published:** 2024-02-14

**Authors:** M G Sedeuil, A Gonneaud, V Giroux

**Affiliations:** Biologie cellulaire, Universite de Sherbrooke, Sherbrooke, QC, Canada; Biologie cellulaire, Universite de Sherbrooke, Sherbrooke, QC, Canada; Biologie cellulaire, Universite de Sherbrooke, Sherbrooke, QC, Canada

## Abstract

**Background:**

Colon cancer affects 1 in 15 people and is the 3^rd^ deadliest cancer worldwide. The standard of care consists of surgical resection combined with 5-fluorouracil (5-FU) chemotherapy. However, relapses remain frequent due to a partial or complete loss of sensitivity to treatment, also known as chemoresistance. Over the last decades, studies have revealed the importance of intratumoral heterogeneity in this phenomenon, and in particular of two components: cancer stem cells (CSCs) and the microenvironment. CSCs are cells with a high capacity of self-renewal, potency (ability to give rise to different cell types) and resistance to anti-cancer treatments. The microenvironment, for its part, comprises all the components surrounding tumour cells, such as cancer-associated fibroblasts (CAFs). Although their respective importance in resistance has been well studied, it is important to better understand their respective modulation during resistance as well as their interactions.

**Aims:**

Identify the specific changes occurring in CSCs and CAFs, as well as their interactions, during the induction of 5-FU resistance in colon cancer.

**Methods:**

To achieve this goal, we are establishing 5-FU resistant models using organoid lines from colon cancer patients. First, we validated that parental organoids maintain their expected cellular heterogeneity by immunofluorescence. We also confirmed that our parental organoids are sensitive to 5-FU by measuring the survival through a WST1 assay.

**Results:**

Indeed, we showed the presence of CSCs (ALDH1+) and proliferative cells (Ki67+), among others. With a baseline IC50 of 5µM, we concluded that they can be used to generate a 5-FU resistant line. In addition, our initial findings suggest that the conditioned media of CAF cultures decreases the sensitivity of parental organoids to 5-FU, emphasizing the need to study deeper the interaction between these two tumor components

**Conclusions:**

In summary, our preliminary results suggest that the interaction between tumor cells and CAFs modulates the response to chemotherapy. We are currently exploring whether CSCs are particularly involved in this phenomenon. In addition, we are developing patient-derived xenograft models (PDX) sensitive or resistant to 5-FU in order to study in vivo the interactions between CAFs and CSCs. At the end, this project will provide a better understanding of the changes required for resistance, paving the way for new targeted therapies

**Funding Agencies:**

Canada foundation for innovation, Canada Research Chairs

